# Kam Sweet Rice (*Oryza sativa* L.) Is a Special Ecotypic Rice in Southeast Guizhou, China as Revealed by Genetic Diversity Analysis

**DOI:** 10.3389/fpls.2022.830556

**Published:** 2022-03-07

**Authors:** Chunhui Liu, Di Cui, Aixia Jiao, Xiaoding Ma, Xiaobing Li, Bing Han, Huicha Chen, Renchao Ruan, Yanjie Wang, Longzhi Han

**Affiliations:** ^1^Institute of Crop Sciences, Chinese Academy of Agricultural Sciences, Beijing, China; ^2^College of Life and Environmental Sciences, Minzu University of China, Beijing, China; ^3^Institute of Crop Germplasm Resources, Guizhou Academy of Agricultural Sciences, Guiyang, China

**Keywords:** Kam Sweet Rice (KSR), genetic resource, haplotypes, bacterial blight, rice blast

## Abstract

Kam Sweet Rice (KSR) is a special kind of rice landrace that has been domesticated for thousands of years by the local Dong people in southeast Guizhou province, China. KSR has many distinguishing characteristics including strong fragrance; high resistance to diseases, pests, and adverse abiotic conditions; difficulty of threshing; and glutinous texture. There is a lack of systematic research on its genetic diversity. In this study, we analyzed the levels and patterns of genetic diversity and nucleotide variation in 1,481 rice germplasm using simple sequence repeat (SSR) markers and single nucleotide polymorphism (SNP) haplotype analysis of six unlinked nuclear loci. The accessions included 315 KSR resources from southeast Guizhou, 578 rice landraces from six rice-growing ecological zones in Guizhou, 546 rice landraces from nine provinces around Guizhou, and 42 wild rice sources. Genetic diversity and heterozygosity of KSR were both low, and thus KSR might be close to a pure rice line. Population structure analysis showed that KSR was isolated into a single type of rice, which had a large genetic distance and a unique genetic background compared to the local varieties in Guizhou province, indicating that KSR is a special rice ecotype. Haplotype analysis of the target genes showed that the population of KSR was rich in haplotypes for resistance to bacterial blight (*Xa23*) and rice blast (*Pid3*), and identified unique haplotypes that were different from those of the six rice ecotypes in Guizhou. This study shows that KSR is an excellent rice germplasm resource, provides important information for the improvement and utilization of rice landraces, and serves as a reference for formulating effective rice conservation measures.

## Introduction

Rice (*Oryza sativa* L.), one of the three major global food crops, has a long history of domestication and is now the staple food of nearly half of the world’s population. Rice germplasm resources are the material basis supporting original innovation of agricultural science and breeding (Liu et al., 2018), as well as strategic resources to ensure food, ecological, and seed industry security (Han and Cao, 2005; Liu, 2019). As products of long-term natural and artificial selection in the process of rice cultivation and domestication, rice landraces are genetically diverse and contain many excellent genes for resisting disease and stress, and for high quality and high yield (Pusadee et al., 2009; Cui et al., 2016). Landraces have high utilization value for improving rice yield, disease resistance, and quality (Meyer et al., 2016; Dong et al., 2020). Rice genetic diversity is the basis of variety improvement and is essential for its production. However, with the continuous progress of breeding technology, some excellent varieties have been widely promoted, resulting in variety simplification, accompanied by loss of numerous genes and reduced crop genetic diversity (Qi et al., 2006). Therefore, landrace germplasm resources have gradually become vital materials for breeders to select breakthrough varieties and explore high-quality genes due to their unique regional adaptability and rich genetic diversity.

China has a long history of rice farming and rich landrace resources (Li et al., 2001; Han et al., 2002; Gao, 2003), especially in Yunnan, Guangxi, and Guizhou Provinces (Wang et al., 2016; Zeng et al., 2017; Cui et al., 2020), where ethnic minorities live in tight-knit communities in southwest China. Guizhou province is rich in rice landrace resources, ranking fourth in the number of landraces in China, after Guangxi, Guangdong, and Yunnan provinces (Han and Cao, 2005; Ruan et al., 2007). Rice landraces in Guizhou have undergone long-term natural evolution and artificial selection, forming varieties with diverse ecological types that have outstanding resistance to diseases and pests (Ruan et al., 2015). Among them, the “HE” resource is a traditional glutinous rice variety cultivated by the Dong people for thousands of years (Supplementary Figure 1). It is suitable for growing in cold areas such as mountains and valleys and in areas with poor soil fertility, and has strong resistance to disease, pests, and stress (Wu et al., 2010; Zhou et al., 2020). Kam Sweet Rice (KSR) is the Dong people’s common name for this resource. The only concentrated planting area of KSR in the world occurs in Congjiang, Liping, and Rongjiang counties of southeast Guizhou Province.

Grains of KSR are plump and milky white. Steamed KSR has many outstanding characteristics including softness, even with cold rice; stickiness; strong fragrance; and a good nutritional profile (Wang Y. J. et al., 2018). Therefore, KSR is the staple food of the Dong people as well as the raw material for processing various non-staple foods. We analyzed previous studies, and found that KSR has low amylose content. The variation ranged from 0.69 to 2.81% (Li et al., 2019; Shen et al., 2021; Zhang et al., 2021), with an average of 1.55%, which was significantly lower than the *japonica* varieties approved in Guizhou province in the last 10 years (16.2%) (Yu et al., 2019) and the excellent *japonica* varieties in China (16.72%) (Ma et al., 2021) (*P* < 0.01). The low amylose content is one of the key factors for the stickiness and good taste of KSR, and Lai (1988) even found that some KSR varieties contain almost no amylose. Gel consistency, alkali spreading value, and fat content are also key indexes to determine rice taste quality (Bao, 2012; Shi et al., 2019). Jia (1988) compared KSR with other glutinous rice in Guizhou and found that the gelatinization temperature (alkali spreading value method) of KSR was 65.7°C, lower than that of general glutinous rice (67.1°C). KSR thus has a high alkali spreading value, resulting in its soft and desirable rice quality and good taste. The fat content of KSR (3.66%) was higher than that of other glutinous rice in Guizhou (3.00%), and also higher than that of the high-quality *indica* (2.71%) and *japonica* (2.85) screened in China (Yu et al., 2007). Generally speaking, increasing fat content could significantly improve flavor and texture of rice (Yu et al., 2006). Overall, KSR has lower amylose content, lower gelatinization temperature, and higher fat content with better quality characteristics than other rice varieties.

From 2013 to 2015, we investigated the cultivation, traditional utilization, and biological characteristics of KSR in 33 Dong villages, and collected 168 KSR resources, which are preserved in the National Gene Bank (Wang Y. J. et al., 2018). We found that KSR is not only the staple food of the Dong people, but is also fully utilized in their traditions, such as food culture, festival celebrations, and religious beliefs. Dong people believe that KSR tastes better than hybrid rice, and it is thus indispensable to many foods in their daily life. For instance, KSR is steamed as a staple food, fried KSR is used for snacks, glutinous rice wine and KSR oil tea is used for beverages. KSR is also combined with ingredients to pickle fish, meat, and vegetables. KSR is a necessity for the Dong people to celebrate festivals and visit relatives and friends. For the Dong people, KSR is the most precious rice variety and is given to others as a gift for a newborn, when young people get married, and for other important traditional festivals. In addition, KSR is necessary in some sacrificial ceremonies of the Dong people. For these rites, KSR cannot be replaced by other rice or hybrid rice varieties, due to the specific requirements of the sacrificial rites to use local traditional rice. For example, when Dong people attend funerals, they always carry KSR. In the traditional production of the Dong people, stalks of KSR are often used as weaving materials to make brooms and containers for storing things. In addition, stalks of KSR that have been burned to ashes are often used for dyeing cloth.

Due to its excellent characteristics and its utilization by the Dong nationality, it has attracted extensive attention from scholars at home and abroad since the 1980s (Stone, 2008), and was named a “Specialty Rice” (Bedigian, 2003) by the Food and Agriculture Organization (FAO) of the United Nations. KSR not only enriches human rice cultivation, but also enriches the rice germplasm resources in China. The genes controlling the desirable traits of KSR are rare and important genetic resources, making southeast Guizhou an important gene bank of rice varieties. Some scholars have analyzed the variety collection and naming of KSR (Lei et al., 2021), and the genetic diversity within the KSR population (Ma et al., 2010), laying a good foundation for our follow-up research. However, the experimental materials in previous studies were relatively limited and confined to a small number of KSR populations, and there was a lack of comparative studies with other local rice varieties.

Due to the influence of foreign cultures and the widespread cultivation of hybrid rice and high-efficiency cash crops, the area of traditional rice cultivation in China has declined sharply. Our previous research showed that the planting area and variety number of KSR decreased sharply (Wang Y. J. et al., 2018), and relevant results of Lei et al. (2021) were also consistent with this. The number of KSR varieties in southeast Guizhou has decreased from more than 300 in the 1980s to more than 100 currently. Therefore, it is urgent to protect and study these rare landrace varieties. This study, based on our team’s previous research (Wang et al., 2016; Wang Y. J. et al., 2018), used modern molecular markers to analyze the genetic diversity, population structure, and six domestication gene haplotypes of 1,481 rice landrace varieties, KSR, and wild rice materials from ten provinces, aiming to provide important information for the improvement and utilization of local rice varieties, and provide a reference for implementing effective protection measures.

## Materials and Methods

### Plant Materials

This study was performed at a large scale using 1,481 accessions from Guizhou and surrounding provinces. We divided 1,481 accessions into four groups, namely, KSR, rice landraces of Guizhou province (GZ), rice landraces of neighboring provinces (NP), and wild rice (WR). Among them, 315 KSR were collected (including 147 from the National Gene Bank and 168 collected by our team in southeast Guizhou in 2015); for GZ, 578 rice landraces distributed in 72 counties and six ecological rice-growing areas from other areas of Guizhou except southwest were selected; 546 NP accessions were selected from Jiangsu, Zhejiang, Anhui, Jiangxi, Guangdong, Guangxi, Hunan, Hubei, and Yunnan; and 42 WR accessions were selected from Guangdong, Guangxi, and Hunan. Furthermore, the 1,481 materials included 537 core germplasms from the primary core germplasm of cultivated rice in China, to represent rice grown throughout China. Rice landraces in Guizhou covered all ecological and climatic types in the province. Rice landraces from all of the surrounding provinces of Guizhou were also taken, and counties in these provinces were sampled as much as possible to achieve comprehensive coverage. The sources and quantities of 1,481 accessions are shown in Supplementary Table 1.

### DNA Extraction, Simple Sequence Repeat Genotyping

Total genomic DNA was extracted manually from fresh young leaves using a modified CTAB procedure (Doyle and Dickson, 1987). Fresh leaves stored at ultra-low temperature were taken, ground and added into a 2.0 ml centrifuge tube with CTAB 750 μl. The mixture was incubated in water bath at 65°C for 30 min. centrifuged at 12,000 rpm for 5 min. The supernatant was transferred to a new centrifuge tube, added with 300 μl of chloroform-isoamyl alcohol (24:1) mixture. Centrifuge at 12,000 rpm for 5 min again, 300 μl of supernatant into a new centrifuge tube. Added with an equal volume of isopropanol, shaken and put at −20°C for 60 min, and centrifuged at 12,000 rpm for 5 min. The supernatant was carefully discarded, and the precipitate was washed with 70% ethanol twice, centrifuged for 30 s after each wash. After the ethanol was removed, the DNA precipitate was dried at room temperature, dissolved in 200 μl ddH_2_O and finally preserved at −20°C.

A set of 36 SSRs (Supplementary Table 2), evenly distributed throughout the rice genome, was used to analyze population structure. SSRs were amplified using a polymerase chain reaction (PCR) with fluorescently labeled primers in a 15-μL reaction volume, which is the same as Cui et al. (2016). PCR products were size separated on a 3730XL DNA Sequencer equipped with GENESCAN software (ABI, United States). Fragment lengths were analyzed using Gene Marker V1.6 (Soft Gene), and the data were retained for later analysis.

### Population Structure and Differentiation

Sorting the peak values obtained by capillary electrophoresis. Method of molecular marker data conversion, statistical analysis, Nei’s genetic distance calculation, analysis of molecular variance (AMOVA) and population differentiation coefficient calculation refer to Han et al. (2019).

### Sequencing, Sequence Analysis, and Neutrality Test

According to the characteristic of KSR described by the Dong farmers in local research and existing research results (Chen et al., 1999; Zheng et al., 2010), KSR is more resistant to cold, bacterial blight and brown planthopper, but the specific gene and mechanism are not clear, which are only limited to identification experiments. Combined with relevant cloned genes that have been reported (Chen et al., 2011; Wang et al., 2014, 2015), genes used in this study represent six unlinked nuclear loci across the rice chromosomes (*SKC1*, *SAP8*, *Pid3*, *Xa23*, *GS5*, and *Ehd1*). The structure of the gene is showed in Supplementary Figure 2 (Hu et al., 2015), and detailed information about the genomic location, gene function and primer sequence of the target gene is shown in Supplementary Table 3.

The DNA sequences of parts of six rice genes (*SKC1*, *SAP8*, *Pid3*, *Xa23*, *GS5*, and *Ehd1*) were obtained from the NCBI database, and oligonucleotide primers were designed using primer5. PCR amplifications are the same as Han et al. (2019). PCR products were Sanger sequenced, and data were retained for later analyses. DNA sequence analysis and neutrality test refer to Cui et al. (2016). The number of isolated loci (S), haplotype number (H), haplotype diversity (Hd), mean variation of paired loci (π), and Watterson estimation parameter (θw) were calculated.

### Phylogene Network Analysis

Haplotype networks were constructed by mutational steps using Network 4.5 (Bandelt et al., 1999). These networks represent the genetic distance of DNA sequences or alleles and are mainly composed of circles of different size and color, and lines linking the circles. The circle size is proportional to the number of samples within a given haplotype, while the lines between haplotypes represent mutational steps between the alleles. The numbers next to the circle represent the haplotype number. Circle color represents different collection times of rice landraces. If more than one nucleotide difference exists between linked haplotypes, it is indicated by numbers next to the lines. Because many haplotypes were obtained for the nuclear loci, only major haplotypes, containing more than three individuals each, were selected for construction of the networks.

## Results

### Genetic Diversity and Genetic Differentiation

In total, 36 pairs of SSR markers were selected to detect the genetic diversity of the population, as shown in Supplementary Table 4. A total of 1,021 alleles were detected in 1,481 accessions, and the number of alleles at each locus ranged from 9 (RM495) to 63 (RM592), with an average of 28.36. The alleles of RM592, RM206, RM257, RM219, and RM228 were abundant, numbering 63, 60, 47, 47, and 46, respectively. The genetic diversity indexes of 36 SSR loci in 1,481 materials varied from 0.5185 (RM495) to 0.9591 (RM206), with an average of 0.8026. The heterozygosity (He) ranged from 0.0621 to 0.4882, with an average of 0.228. Polymorphic information content (PIC) values ranged from 0.4124 to 0.9576 with an average of 0.7815.

The assessment of genetic diversity in the four groups of rice accessions is shown in Table 1. WR had the highest gene diversity index, He, and PIC value, which were 0.8526, 0.5833, and 0.8374, respectively. NP had the highest average number of alleles (24.03), and had a relatively high gene diversity index and PIC value of 0.8088 and 0.7870, respectively, which were higher than the average level of all materials. The genetic variation and He rate of KSR were low, indicating that KSR was domesticated by the Dong people for a long time, and may be close to a pure line. AMOVA results showed that the genetic differences between and within populations were extremely significant (*P* < 0.001), at 6.01 and 93.99%, respectively, indicating that the genetic differences mainly came from within populations.

**TABLE 1:** Summary statistics for genetic diversity in KSR, GZ, NP, and WR group.

Subgroup	Sample size	Major allele frequency	Alleles	Alleles/locus	Gene diversity	Heterozygosity	PIC
KSR	315	0.4285	560	15.56	0.6993	0.1010	0.6717
GZ	578	0.3699	703	19.53	0.7635	0.1491	0.7367
NP	546	0.3124	865	24.03	0.8088	0.3575	0.7870
WR	42	0.2414	565	15.69	0.8526	0.5833	0.8374
Total	1481	0.3213	1021	28.36	0.8026	0.2280	0.7815

### Population Structure and Differentiation

**FIGURE 1:**
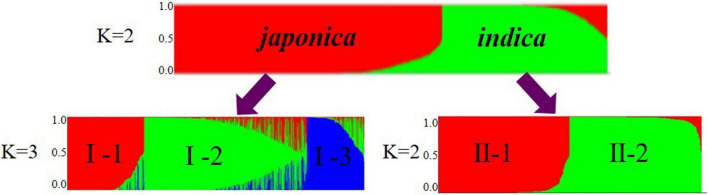
Model-based cluster membership for 1481 in *indica* and *japonica* groups.

To infer population structure, we performed model-based simulations for the 1,481 accessions using 36 SSRs. When *K* = 2, the statistical value ΔK showed a peak, suggesting that the number of subpopulations should be two, namely, *japonica* (Group I) and *indica* (Group II) (Figure 1). Further analysis of the two groups showed that Group I was further divided into three subgroups named I-1, I-2, and I-3. Among them, I-1 had 218 rice accessions, 94.5% of which are *japonica*-KSR; I-2 had 447 rice accessions, which are *japonica*-NP and a few *japonica*-GZ; and I-3 had 116 rice accessions, which are most of the *japonica*-GZ. These results indicate that KSR is different from GZ, and forms a separate category with a unique genetic structure. Group II of the *indica* rice subgroup was subdivided into II-1 and II-2 subgroups. II-1 had 262 rice accessions containing *indica* GZ and small number of *indica*-KSR (accounting for 15.9% of all KSR samples), indicating that KSR were mostly *japonica*. II-2 had 268 rice accessions that were *indica*-NP and WR.

**FIGURE 2:**
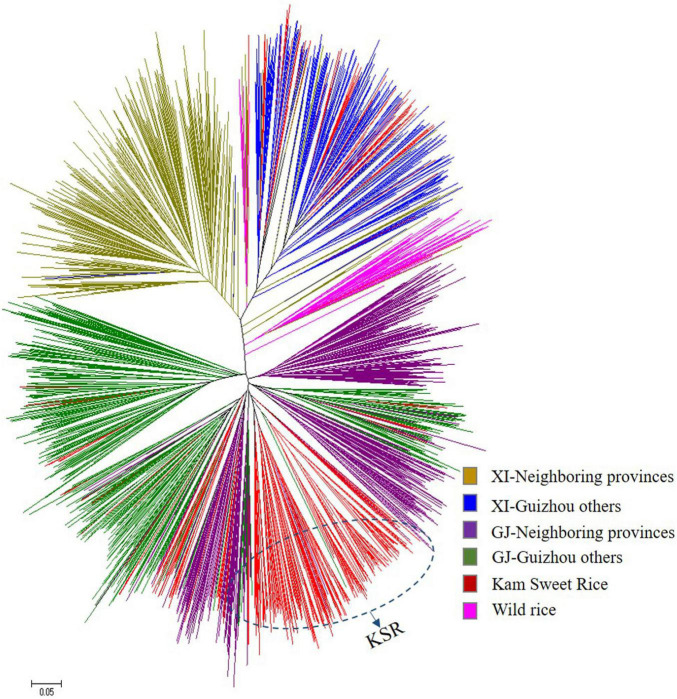
Unrooted neighbor-joining trees of 1481 accessions based on Nei’s genetic distance.

To further study the population structure of the 1,481 rice varieties, we constructed an N-J cluster diagram based on Nei’s genetic distance (Figure 2). The 1,481 accessions were divided into *indica* and *japonica* subpopulations and wild rice subpopulations. The *indica* population was further divided into two groups and the *japonica* population into three groups. The results of N-J clustering and the structure analysis based on the model were consistent, indicating that most KSR germplasm was *japonica*, which was different from other rice resources in Guizhou, though all of them originated from the same province. Rice resources from other parts of Guizhou are mixed and clustered together, but all KSR accessions were clustered together, highlighting its unique genetic background, and identifying KSR as a special ecotypic rice species.

**FIGURE 3:**
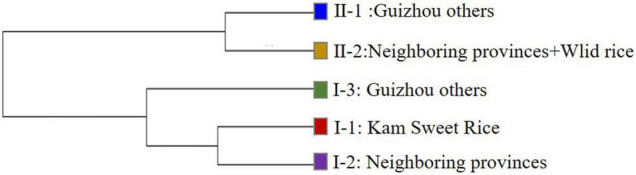
N-J clustering dendrogram based on Nei’s genetic distance.

AMOVA results showed that the genetic differences among and within these five subpopulations were 21.43% and 78.57%, respectively (*P* < 0.001), indicating that the genetic differences mainly came from within subpopulations, and the average coefficient of genetic differentiation between subpopulations was 0.2201 (*P* < 0.001), with a range of 0.0985–0.3198. We calculated the genetic distance and differentiation coefficients of five subpopulations based on Nei’s minimum distance (1972) using Power Marker3.25 and Arlequin 3.5.2.2, respectively (Table 2). The results showed that the genetic distance and differentiation coefficients of different subpopulations had the same trend. The genetic distance between KSR (I-1) and NP (I-2) (0.0787) was less than that of GZ (I-3) (0.1670) in the *japonica* subgroup. Moreover, the FST value (FST_*KSR/GZ*_ = 0.2308, FST_*KSR/NP*_ = 0.1141) showed the same result, indicating that KSR has a large genetic differentiation (0.15 < 0.2308 < 0.25) from GZ, marking it as a special ecotypic rice variety different from the local rice varieties in Guizhou province. On the contrary, KSR had small genetic differentiation and a close relationship with NP, suggesting that there may be a certain evolutionary relationship between them. The result of N-J clustering dendrogram based on Nei’s genetic distance is the same (Figure 3).

**TABLE 2:** Genetic distances as measured by Nei’s minimum distance (above diagonal) and pairwise Fst comparisons (below diagonal) between different subgroups.

	I-1	I-2	I-3	II-1	II-2
I-1		0.0787	0.1670	0.2815	0.2577
I-2	0.1141**		0.1015	0.2269	0.1912
I-3	0.2308**	0.1291**		0.2729	0.2522
II-1	0.3198**	0.2458**	0.3046**		0.0726
II-2	0.2839**	0.2059**	0.2689**	0.0985**	

**Significance level P < 0.001.

### Nucleotide Diversity and Neutral Test

**FIGURE 4:**
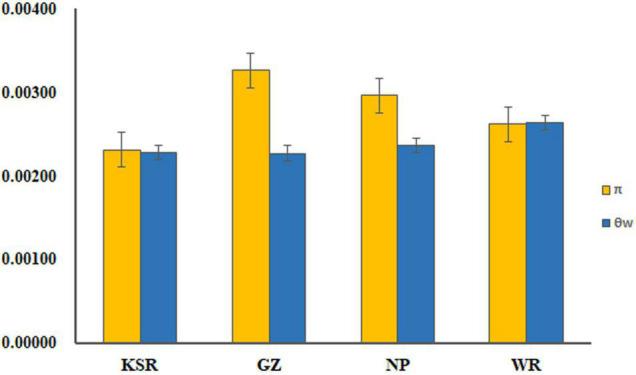
Mean nucleotide variation in KSR, GZ, NP, and WR groups.

Six unlinked gene regions–*SKC1*, *GS5*, *Pid3*, *SAP8*, *Ehd1*, and *Xa23*–were sequenced in 1,481 accessions, and the gene structures of these nuclear loci are shown in Supplementary Figure 2. Chromosome distribution of SSR loci and six target genes are showed in Supplementary Figure 3. The length of aligned sequence for each locus ranged from 482 to 670 bp, with a total length of 3,700 bp. The standard statistics of sequence polymorphisms for each locus are summarized in Supplementary Table 5. The term π indicates nucleotide diversity and θw represents diversity of segregating sites. There was no significant difference (*P* > 0.05, for both π and θw) of mean nucleotide variation among KSR (π = 0.0023, θw = 0.0023), GZ (π = 0.0033, θw = 0.0023), NP (π = 0.0030, θw = 0.0024), and WR (π = 0.0026, θw = 0.0026) (Figure 4). The neutral test included Tajima’s *D* value, Fu and Li’s D* value, and F* value of each locus (Supplementary Table 5). There were no significant differences in the neutral test values for most nuclear genes except for a few loci. *D* values of *SKC1*, *GS5*, *Pid3*, and *SAP8* in the KSR population were negative, indicating a large number of low-frequency alleles (rare gene loci) on these genes, which control salt tolerance, grain size, rice blast resistance, and cold tolerance in rice. This may be due to negative selection or strong positive selection during the long-term domestication by the Dong people.

### Network Relationships of Haplotypes From Six Domestication Genes

**FIGURE 5:**
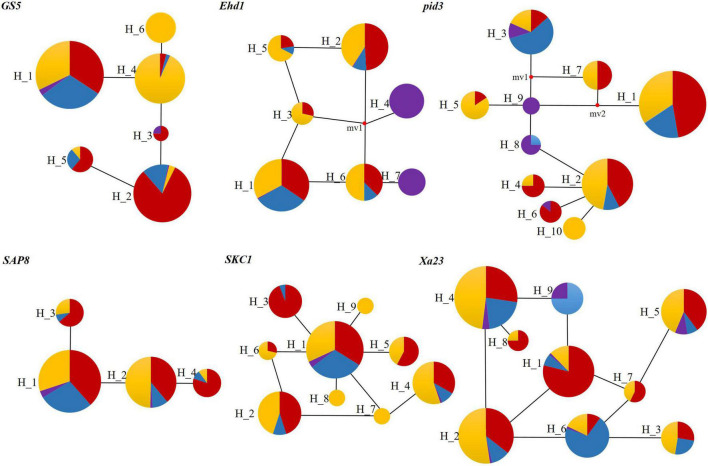
Six loci’s haplotype networks in KSR, GZ, NP, and WR groups of rice landraces. The circle size is proportional to the quantity of the samples within a given haplotype, and the numbers next to the circle represent the haplotype type. Lines between different haplotypes represent the mutational steps between alleles. Circle color represent different rice variety: Blue—KSR; Red—GZ except for KSR; Yellow—NP; Purple—WR.

**FIGURE 6:**
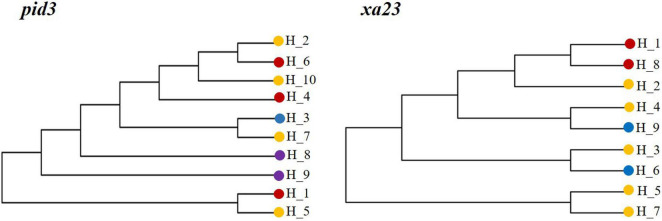
Haplotypes of *Pid3* and *Xa23* gene based on NJ cluster map.

We investigated the network of major haplotypes at six loci (Figure 5), with all key haplotypes for each gene shown on branches. There were 6, 7, 10, 4, 9, and 9 haplotypes at *GS5*, *Ehd1*, *Pid3*, *SAP8*, *SKC1*, and *Xa23* gene loci, respectively. Among the six genes sequenced, KSR, GZ, NP, and WR shared dominant haplotypes H_1 and H_2, which appeared most frequently, and other haplotypes were radiating or staggered around them. The haplotype frequencies were as follows: GZ (39.0%) > NP (36.8%) > KSR (21.3%) > WR (2.6%) (Supplementary Table 6). NP had unique haplotypes H_6, H_10, and H_7, H_8, and H_9 of *GS5*, *pid3*, and *SKC1* genes, but H_4, H_7, and H_9 were unique haplotypes of wild rice in *Ehd1* and *pid3* genes. Eight haplotypes (H_1 of *Ehd1*; H_1 of *GS5*; H_3 of *Pid3*; H_1 of *SAP8*; H_1 of *SKC1*; and H_3, H_6, and H _9 of *Xa23*) were common (frequency > 25%) in KSR. Further, three haplotypes (H_3 of *Pid3*, H_6 and H _9 of *Xa23*) were dominant in KSR, with proportions of 56.8, 70.5, and 75.0%, respectively. This indicated that genes for resistance to blast and bacterial blight were abundant in KSR, consistent with the nucleotide diversity and the neutral test of the six genes. Subsequently, we performed haplotype cluster analysis of *Pid3* and *Xa23* genes (Figure 6), which showed that the dominant KSR haplotypes (H_3 of *Pid3*, H_6 and H_9 of *Xa23*) and the dominant NP haplotypes (H_7 of *Pid3*, H_3 and H _4 of *Xa23*) clustered into one group. These results suggested that KSR might have a close evolutionary relationship with NP, consistent with the clustering results based on SSR molecular marker data.

AMOVA (Table 3) showed that the genetic differences at the six loci were mainly within populations, with the mean variation accounting for 90.92% and the amplitude of variation ranging from 82.16 to 95.96%, consistent with the results based on SSR data analysis.

**TABLE 3:** Analysis of molecular variance for KSR, GZ, NP, and WR groups.

Locus	Source of variation	Sum of squares	Variance components	Percentage of variance (%)
*GS5*	Among populations	257.189	0.2575	17.84
	Within populations	1751.113	1.8559	82.16
*Ehd1*	Among populations	114.596	0.1134	10.73
	Within populations	1392.447	0.9434	89.27
*SAP8*	Among populations	20.284	0.0199	7.82
	Within populations	345.742	0.2341	92.18
*Pid3*	Among populations	149.567	0.1469	9.15
	Within populations	2161.612	1.4586	90.85
*Xa23*	Among populations	51.615	0.0434	4.88
	Within populations	1495.273	0.8467	95.12
*SKC1*	Among populations	26.089	0.0245	4.04
	Within populations	866.200	0.5825	95.96
Average	Among populations	103.223	0.1009	9.08
	Within populations	1335.398	0.9869	90.92

### Analysis of Geographical Distribution of Haplotypes

**FIGURE 7:**
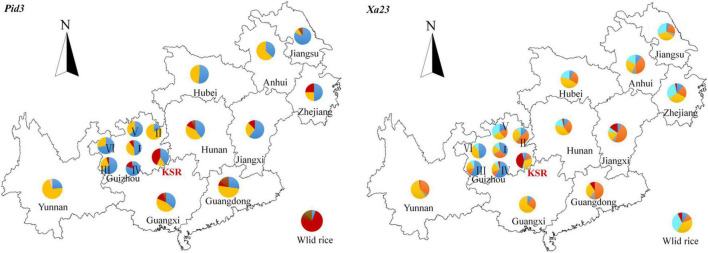
A map showing the distribution of haplotypes. Pie charts show the proportions of the haplotypes within each provinces. Haplotypes are indicated by different colors.

To compare the geographical distributions of haplotypes, we divided all accessions into 17 populations according to the provenance of different provinces (accessions from Guizhou province were divided into six ecological regions according to the provenance of different ecological regions). For *Pid3* (Figure 7), the dominant haplotype of KSR is H_3 (red in the figure), which is similar to that of wild rice, while the dominant haplotype of other populations is H_1 or H_2 (blue or yellow in the figure). For *Xa23* (Figure 7), the dominant haplotype of KSR is H_6 (red in the figure), which is different from all other groups, indicating that KSR had superior haplotypes in blast resistance and bacterial blight resistance. In addition, the haplotype composition of KSR is similar to NP, especially in Guangxi, Guangdong, Hunan, Jiangxi, etc., but different from Guizhou province, and it is consistent with the results of haplotype clustering based on four populations.

## Discussion

### Comparison of Genetic Diversity Based on Simple Sequence Repeat

Southwest China is one of the largest centers of genetic diversity of cultivated rice in China and even globally. Guizhou possesses a very large gene pool of rice germplasm resources with 9,000 accessions including rice landrace varieties, upland rice, KSR resources, and other breeding varieties (Ruan et al., 2015). Many varieties have excellent traits such as fast growth, high yield, insect resistance, large seeds, and good quality, but the utilization rate of whole rice resources is low (Zhang et al., 2007).

In this study, 36 pairs of SSRs were used to detect the genetic diversity of 315 KSR accessions from southeast Guizhou province, 578 rice landraces from Guizhou province, 546 rice landraces from neighboring provinces, and 42 wild rice accessions. We detected an average of 28.36 alleles in these 1,481 accessions, and 15.56 alleles of KSR, which was higher than previous studies by other researchers, including 537 rice varieties in Guizhou Province (12.08 alleles per locus) (Zhang et al., 2007), 40 rice landraces currently cultivated in Yunnan Province (4.46 alleles per locus) (Xu et al., 2011), 50 local and improved varieties in India (12.47 alleles per locus) (Kumbhar et al., 2015), 64 rice landraces in an alpine region of India (8.49 alleles per locus) (Roy et al., 2015), and 314 rice varieties in northern Laos (3.49 alleles per locus) (Muto et al., 2019). The mean value of polymorphic information content (PIC) of 1,481 accessions was 0.782 slightly higher than 50 Indian rice varieties (0.759) detected by Kumbhar et al. (2015) using 15 pairs of SSRs, 91 rice varieties from eastern and northeastern India (0.747) detected by Das et al. (2013) using 23 pairs of SSRs, and significantly higher than 1,610 wild rice accessions from Guangxi (0.177) tested by 25 pairs of SSR markers (Su et al., 2017). Compared with previous studies, the genetic diversity indexes and PIC values in this study were higher. This may be due to the wide range of material sources, and complex genetic background and structure, which provide valuable information for rice breeding, collection and management of germplasm resources, and rice gene development.

In terms of KSR diversity, Lei et al. (2021) used 20 pairs of SSR markers to detect the genetic diversity of 95 KSR, among which 8 pairs of SSR markers were the same as those in our study, and the selection principles of SSR markers were basically similar between these two researches. However, the diversity of KSR were slightly different. In our study, the average allelic variation (15.56), gene diversity index (0.6993) and PIC (0.6717) of 315 KSR were higher than those of 95 KSR of Lei et al. (2021) (6.4, 0.6061, and 0.5576). This result indicates that sample size has direct influence on genetic diversity. In addition, comparing the genetic diversity of KSR, GZ, NP, and WR, the results showed that the gene diversity index (0.6993), heterozygosis (0.1010), and PIC (0.6717) of KSR were all lower than those of other populations. These diversity parameters in Lei et al.’s (2021) study were also lower than GZ, NP, and WR, indicating that the genetic variation in KSR population is small, and it is a special variety bred by the Dong people for a long time in a specific region and under a specific traditional cultural background. However, KSR is close to pure line after long-term selection, which may bring genetic vulnerability. It is very important to evaluate the genetic diversity of KSR for gene preservation and selection of superior varieties, low genetic variation is closely related to its life history, ecological characteristics and narrow distribution range. Therefore, it is necessary for us to have a deeper understanding of the diversification process of KSR under the influence of Dong traditional culture and management mode, so as to rationally protect and utilize this rare rice resource.

### Genetic Structure of Rice Landraces From South of the Yangtze River

Cluster analysis and population structure analysis are effective means to explore the genetic diversity and background of crop germplasm resources, and are prerequisites for the protection and utilization of crop germplasm resources (Wang W. S. et al., 2018). Many scholars have studied the population structure differentiation of cultivated rice at the gene level by using DNA molecular markers (Pritchard et al., 2000; Garris et al., 2005; Semon et al., 2005). Studies have shown that south of the Yangtze River Basin in China is one of the origin centers of Asian cultivated rice (Londo et al., 2006; Molina et al., 2011), therefore, we selected rice landraces from almost all provinces south of the Yangtze River basin, to study their genetic structure.

The 1,481 accessions were divided into *indica* and *japonica* subgroups based on Structure analysis and the N-J cluster map. The *indica* and *japonica* populations were further divided into two and three groups, respectively, similar to the study of population structure division of 537 rice varieties in Guizhou Province by Zhang et al. (2007), and the study of 3,024 rice varieties in China by Zhang et al. (2009). Moreover, KSR was obviously separated from other rice species in Guizhou province in the *japonica* subgroup, according to the calculation of Nei’s genetic distance and the FST value; the genetic distance between KSR and GZ (FST = 0.2308) was higher than that between KSR and NP (FST = 0.1141). Wright (1978) proposed that FST values between 0.15 and 0.25 were high, which indicates that the process of domestication by the Dong people over thousands of years, including their special traditional culture, eating habits, religious customs, and farming methods, caused KSR to differ from other local varieties in the same province. KSR has a unique genetic background and is a special rice ecotype. These results not only provide useful information of evolutionary relationships for genetic breeders using these materials, but also highlight the complexity of genetic structure of germplasm resources, which provides an important reference for selecting hybrid parents of rice landraces.

### Haplotype Analysis Revealed the Excellent Properties of Kam Sweet Rice

Polymorphic variation in gene sequences results in the genetic diversity of species. As the environment changes, some mutation sites that are more adaptable to the environment are retained. The most common sequence variation is the insertion and/or deletion of single nucleotide polymorphisms (SNPs) and InDel fragments (Zhang et al., 2008). SNPs are considered the most frequently occurring genetic polymorphism in plant genomes (Stephens et al., 2001). We sequenced six domestication genes of 1,481 rice accessions and analyzed nucleotide polymorphisms, and found that KSR had numerous rare alleles at the gene loci of disease resistance and cold tolerance. Subsequent studies on haplotypes showed the same result. Haplotypes that no longer allowed adaptation to the local region and the traditional culture of the Dong people were gradually phased out, and instead, new, favorable haplotypes emerged. This is also consistent with the local survey results of our team: farmers generally believed that the excellent resistance to disease and insects of KSR is an important factor for its cultivation (Wang Y. J. et al., 2018). We also found some unique haplotypes of NP and WR (H_6 of *GS5*; H_10 of *Pid3*; H_7, H_8, and H_9 of *SKC1*), which are essential in the breeding of superior varieties.

Therefore, on-farm conservation of farmers’ traditional tillage system is an important way to conserve germplasm diversity, which supplements *ex situ* conservation, and should be encouraged and strengthened, especially in the genetic diversity centers of crops in southwest China.

### Protection of Kam Sweet Rice Genetic Diversity

The genetic diversity of crop germplasm resources is affected by many factors such as genetic population structure, natural selection, and human domestication. Traditional culture is an important factor affecting the diversity of crop varieties in minority areas of China and indigenous and local communities (ILCs) around the world (Xue, 2014; Xue, 2017). Understanding the value of crops is a prerequisite for protecting the resources of crop varieties (Chi et al., 2007). The diversity of local crop varieties is related to cultural customs, and even the dietary habits or taste preferences of different ethnic groups (Bellon, 2004). Farmers in Asia continue to grow thousands of rice varieties with different aromas, tastes, and medicinal and cultural uses to meet their different needs (Bellon et al., 2000; Bertuso et al., 2000; Rijal et al., 2001). For example, farmers use a total of 11 different varieties of corn in their traditional diet in the Oaxaca area of Mexico (Bellon et al., 2003). Additionally, an important reason for maintaining variety diversity of traditional Ethiopian crops such as millet (*Eleusine coracana* L.), wheat (*Triticum turgidum* L.) (Tsegaye and Berg, 2007), and highland barley (*Hordeum vulgare* L.) (Shewayrga and Sopade, 2011) is to meet the needs of local, traditional food cultures.

The Dong people are the descendants of the ancient Yue people, who lived on glutinous rice as their staple food, and as such, the Dong people have a long history of eating glutinous rice. As the most distinctive rice landrace in Guizhou province, KSR not only has good resistance to diseases and insects, but is also used in the traditional diet, festival celebrations, sacrificial activities, and other cultural customs of the Dong people, and thus has been planted and used by the Dong people for thousands of years. In addition, KSR adopts the organic ecological planting mode of “rice-fish-duck” co-existence completely (Wang Y. J. et al., 2018), which has multiple social, ecological, economic, and cultural benefits. These factors improve the yield and quality of rice, drive farmers’ enthusiasm to plant KSR and preserve the precious crop variety resource of the Dong people (Xu, 2010). The traditional culture of the local Dong nationality has thus protected KSR and resulted in it being cultivated and utilized continuously. It is worth noting that the decrease of planting area and variety number of KSR is also affected by environmental conditions, government decisions, and social and economic development. In order to better preserve the genetic diversity of KSR, we need to take effective measures to protect the variety resources of KSR by strengthening the cultural identity of KSR, formulating effective policies, promoting the “rice-fish-duck” symbiotic system, and establishing cooperative agreements between Dong farmers and relevant sales companies.

## Data Availability Statement

The datasets presented in this study can be found in online repositories. The names of the repository/repositories and accession number(s) can be found in the article/Supplementary Material.

## Author Contributions

CL performed statistical analysis and wrote the manuscript. CL, YW, DC, and BH conceived and designed the experiments. YW, AJ, XM, HC, RR, and XL conducted field surveys and collected germplasm resources. YW, AJ, and LH sorted out the collected germplasm resources and incorporated them into the National Medium term Gene bank. YW performed the experiments. LH supervised the thesis. All authors contributed to the article and approved the submitted version.

## Conflict of Interest

The authors declare that the research was conducted in the absence of any commercial or financial relationships that could be construed as a potential conflict of interest.

## Publisher’s Note

All claims expressed in this article are solely those of the authors and do not necessarily represent those of their affiliated organizations, or those of the publisher, the editors and the reviewers. Any product that may be evaluated in this article, or claim that may be made by its manufacturer, is not guaranteed or endorsed by the publisher.
