# Supplementary material for: Kam Sweet Rice (Oryza sativa L.) Is a Special Ecotypic Rice in Southeast Guizhou, China as Revealed by Genetic Diversity Analysis
Source: Front Plant Sci. 2022 Mar 7;13:830556. doi: 10.3389/fpls.2022.830556 (PMC8940365; doi:10.3389/fpls.2022.830556)
Supplement: Supplementary file 1 [file Data_Sheet_1.PDF]

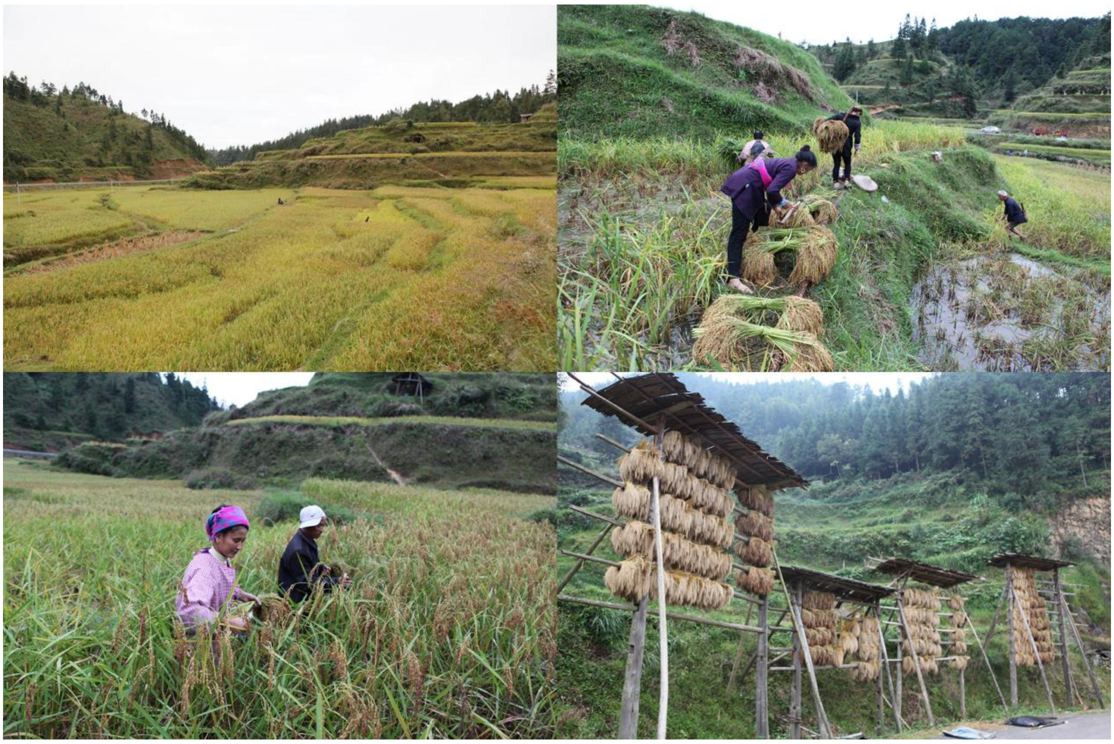

Supplementary Figure S1 Examples of KSR varieties and paddy fields of KSR in Dong villages

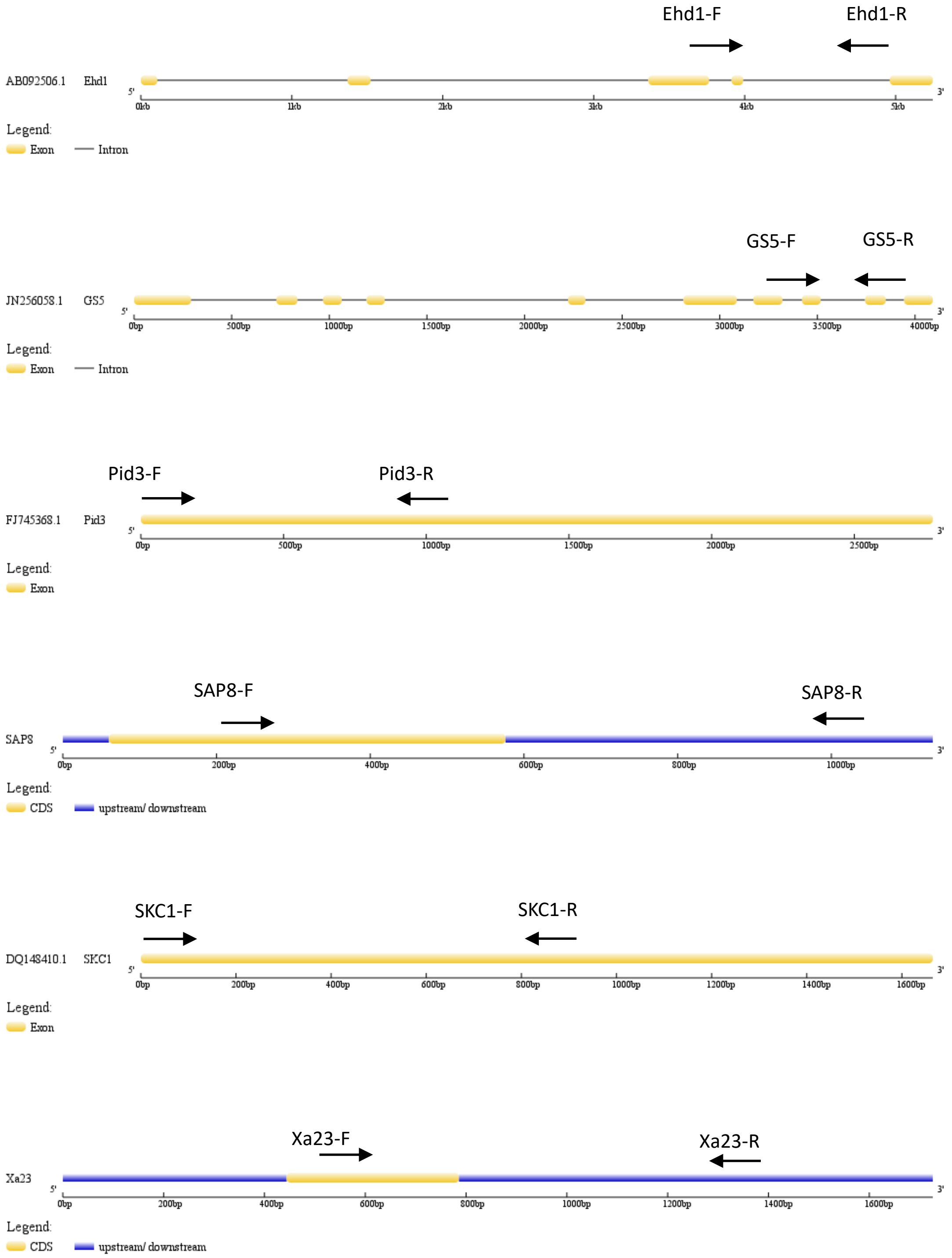

Supplementary Figure 2 Gene structure diagram of six unclear loci

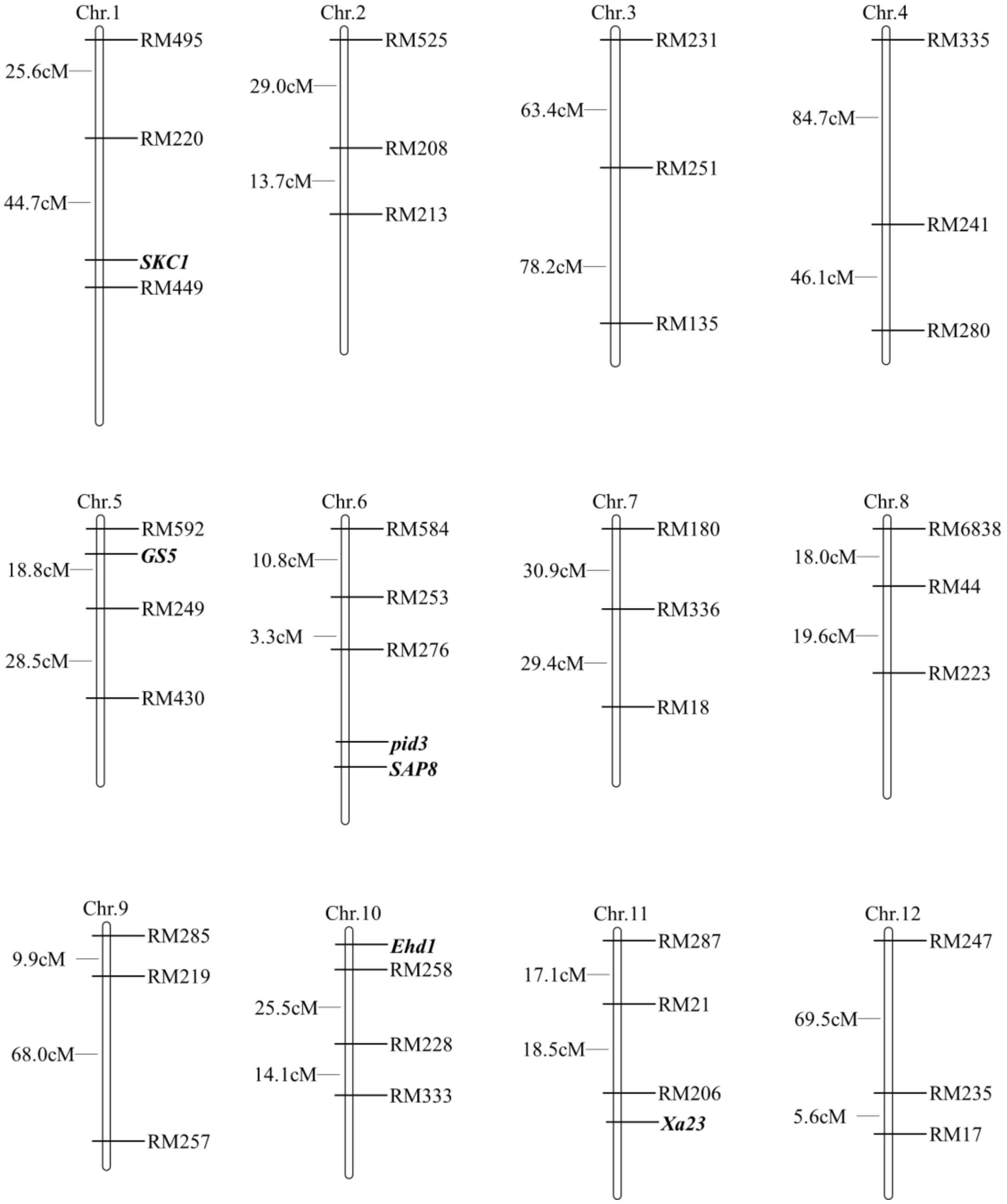

Supplementary Figure 3 Chromosome distribution of SSR loci and six target genes

Supplementary Table S1 Accessions of rice varieties used in the experiment

Population	Source of materials	Number	Subtotal
KSR	Liping, Congjiang, Rongjiang, Jianhe, Jinping and Tianzhu in southeast Guizhou province (6 counties)	315	315
GZ	Guizhou province (72 counties)	578	578
NP	Jiangsu province (34 counties)	57	546
	Zhejiang province (27 counties)	41	
	Jiangxi province (60 counties)	77	
	Guangdong province (42 counties)	47	
	Guangxi province (54 counties)	75	
	Hubei province (44 counties)	63	
	Hunan province (45 counties)	74	
	Anhui province (34 counties)	41	
	Yunnan province (54 counties)	71	
WR	Hunan province	23	42
	Guangdong province	14	
	Guangxi province	5	
Total	1481		

Supplementary Table S2 SSR primers information

Locus	Chromosomes	Forward primer	Reverse primer	Position/cM	Size/bp	Repeat Motif
RM220	1	ggaaggtaactgtttccaac	gaaatgcttcccacatgtct	28.4	100-152	$(CT)_{17}$
RM495	1	aatccaaggtgcagagatgg	caacgatgacgaacacaacc	2.8	117-167	$(CTG)_{7}$
RM449	1	ttgggaggtgttgataaggc	accaccagcgtctctctctc	73.1	106-168	$(AG)_{12}$
RM208	2	tctgcaagccttgtctgatg	taagtcgatcattgtgtggacc	172.7	160-182	$(CT)_{17}$
RM213	2	atctgtttgcaggggacaag	aggtctagacgatgtcgtga	186.4	118-164	$(CT)_{17}$
RM525	2	ggcccgtccaagaaatattg	cggtgagacagaatccttacg	143.7	102-152	$(AAG)_{12}$
RM231	3	ccagattatttcctgaggtc	cacttgcatagttctgcattg	15.7	158-202	$(CT)_{16}$
RM135	3	ctctgtctcctcccccgcgtcg	tcagcttctggccggcctcctc	157.3	111-147	$(CGG)_{10}$
RM251	3	gaatggcaatggcgctag	atgcggttcaagattcgatc	79.1	103-155	$(CT)_{29}$
RM335	4	gtacacacccacatcgagaag	gctctatgcgagtatccatgg	21.5	99-165	$(CTT)_{25}$
RM241	4	gagccaaataagatcgctga	tgcaagcagcagatttagtg	106.2	102-164	$(CT)_{31}$
RM280	4	acacgatccactttgcgc	tgtgtcttgagcagccagg	152.3	128-200	$(GA)_{16}$
RM249	5	ggcgtaaaggttttgcatgt	atgatgccatgaaggtcagc	50.2	96-166	$(AG)_{5}A_{2}(AG)_{14}$
RM592	5	tctttggtatgaggaacacc	agagatccggtttgttgtaa	31.4	235-388	$(ATT)_{20}$
RM430	5	aaacaacgacgtccctgatc	gtgcctccgtggttatgaac	78.7	104-200	$(GA)_{25}$
RM584	6	agaaagtggatcaggaaggc	gatcctgcaggtaaccacac	26.2	158-196	$(CT)_{14}$
RM253	6	tccttcaagagtgcaaaacc	gcattgtcatgtcgaagcc	37	108-160	$(GA)_{25}$
RM276	6	ctcaacgttgacacctcgtg	tcctccatcgagcagtatca	40.3	88-166	$(AG)_{8}A_{3}(GA)_{33}$
RM180	7	ctacatcggcttaggtgtagcaaca	acttgctctacttgtggtgagggact	30.1	107-204	$(ATT)_{10}$
RM336	7	cttacagagaaacggcatcg	gctggtttgtttcaggttcg	61	127-196	$(CTT)_{18}$
RM18	7	ttccctctcatgagctccat	gagtgcctggcgctgtac	90.4	120-196	$(GA)_{4}AA(GA)(AG)_{16}$
RM44	8	acgggcaatccgaacaacc	tcgggaaaacctaccctacc	60.9	92-130	$(GA)_{16}$
RM223	8	gagtgagcttgggctgaaac	gaaggcaagtcttggcactg	80.5	130-172	$(CT)_{25}$
RM6838	8	attaataccgctaccacgcg	tcctcctccacctcaatcac	42.9	100-151	$(TCT)_{14}$
RM219	9	cgtcggatgatgtaaagcct	catatcggcattcgcctg	11.7	148-260	$(CT)_{17}$
RM257	9	cagttccgagcaagagtactc	ggatcggacgtggcatatg	79.7	104-192	$(CT)_{24}$
RM285	9	ctgtgggcccaatatgtcac	ggcggtgacatggagaaag	112.8	128-204	$(GA)_{12}$
RM258	10	tgctgtatgtagctcgcacc	tggcctttaaagctgtcgc	70.8	117-206	$(GA)_{21}(GGA)_{3}$
RM228	10	ctggccattagtccttgg	gcttgcggctctgcttac	96.3	91-183	$(CA)_{6}(GA)_{36}$
RM333	10	gtacgactacgagtgtcaccaa	gtcttcgcgatcactcgc	110.4	129-219	$(TAT)_{19}(CTT)_{19}$
RM287	11	ttccctgttaagagagaaatc	gtgtatttggtgaaagcaac	68.6	89-113	$(GA)_{21}$
RM21	11	acagtattccgtaggcacgg	gctccatgagggtggtagag	85.7	112-168	$(GA)_{18}$
RM206	11	cccatgcgtttaactattct	cgttccatcgatccgtatgg	104.2	116-234	$(CT)_{21}$
RM247	12	tagtgccgatcgatgtaacg	catatggttttgacaaagcg	32.3	120-198	$(GT)_{16}$
RM235	12	agaagctagggctaacgaac	tcacctggtcagcctctttc	101.8	92-134	$(GT)_{24}$
RM17	12	tgccctgttattttcttctctc	ggtgatcctttcccatttca	107.4	122-216	$(GA)_{21}$

Supplementary Table S3 Summary of the genes sequences and the primer sequences used in this study

**Locus**	**Chr.No**	**Aligment length**		**Primer Sequences（5’-3’）**	**Tm（℃）**	**Fountional Association**
		Total	Coding	**Sense /Anti-Sense**		
SKC1	1	613	613	TCATCTCCCTCTTGGGTTTCTTACCTGAACGACACCATCCCT	59	shoot K+ concent 1
GS5	5	620	0	CCTAGCTTGTTTTAAGATGTGGCTCCGTGTTTGCTTGTTGTT	56	regulator of grain size
Pid3	6	667	667	CTATGGCGGAGGGTGTTGTGCAGGTAAGAGCGGGTGGTTT	61	Pyricularia oryzae resistance-d3
SAP8	8	670	279	CATCAATAACTGCGGCTTCTTGAATCCTCACCCAGAGTTTAC	58	stress-associated protein 8
Ehd1	10	482	0	TAACTTATGCGTGCGTGTGCCTCTCATCCTTATCCCCA	56	Early heading date 1
Xa23	11	649	142	TTATTCACCGTTTCCAACAGCCGCATAAACCCCCCTCAAGAAAT	59	Bacterial Blight resistance 23

Supplementary Table S4 Genetic indexes summary of 1481 rice varieties in 36 SSR loci

Marker	Major.Allele. Frquency	GenotypeNo	AlleleNo	Gene Diversity	Heterozygosity	PIC
**RM44**	0.2387	85	21	0.8619	0.1600	0.8478
**RM287**	0.3460	71	16	0.7980	0.1857	0.7739
**RM336**	0.2330	135	31	0.8759	0.2822	0.8648
**RM135**	0.4436	40	13	0.6720	0.1594	0.6173
**RM249**	0.4061	110	35	0.7435	0.1492	0.7091
**RM18**	0.3417	87	27	0.8125	0.2296	0.7927
**RM180**	0.6205	75	36	0.5752	0.1121	0.5441
**RM251**	0.4777	130	27	0.7382	0.2073	0.7221
**RM430**	0.3322	112	39	0.8317	0.1310	0.8169
**RM206**	0.1003	254	60	0.9591	0.3592	0.9576
**RM592**	0.1431	205	63	0.9409	0.3011	0.9379
**RM276**	0.3072	123	30	0.8521	0.1384	0.8400
**RM208**	0.5972	51	12	0.6091	0.3065	0.5839
**RM213**	0.2573	75	20	0.8341	0.2843	0.8138
**RM219**	0.1894	223	47	0.9128	0.3552	0.9070
**RM220**	0.2313	95	25	0.8629	0.2579	0.8496
**RM495**	0.5584	15	9	0.5185	0.0621	0.4124
**RM231**	0.2333	79	22	0.8388	0.2019	0.8191
**RM228**	0.2866	167	46	0.8650	0.2654	0.8541
**RM21**	0.4622	101	30	0.7306	0.2336	0.7056
**RM449**	0.3221	100	29	0.8079	0.2134	0.7851
**RM235**	0.3329	116	28	0.8308	0.1958	0.8156
**RM247**	0.2863	160	44	0.8823	0.2235	0.8753
**RM17**	0.3234	53	21	0.7918	0.1296	0.7647
**RM253**	0.2397	104	24	0.8776	0.1722	0.8667
**RM335**	0.1567	127	25	0.9065	0.2161	0.8992
**RM223**	0.2833	106	21	0.8426	0.2113	0.8263
**RM280**	0.6003	92	30	0.6088	0.2026	0.5861
**RM258**	0.6124	61	17	0.5879	0.2228	0.5593
**RM241**	0.1928	123	29	0.8980	0.3167	0.8897
**RM333**	0.1009	160	31	0.9309	0.4180	0.9266
**RM584**	0.2802	79	22	0.7887	0.2498	0.7581
**RM525**	0.2289	65	19	0.8509	0.2573	0.8341
**RM6838**	0.3133	42	13	0.7442	0.1121	0.7010
**RM285**	0.3565	37	12	0.7688	0.1978	0.7373
**RM257**	0.1300	220	47	0.9436	0.4882	0.9409
**Mean**	**0.3213**	**107.7222**	**28.3611**	**0.8026**	**0.2280**	**0.7815**

Supplementary Table S5 Summary of nucleotide polymorphisms and neutrality tests

Pop.	Gene	L	S	h	Hd	π	θw	D	D*	F*
**KSR**	*SKC1*	613	5	6	0.317	0.0010	0.0013	-0.415	-0.247	-0.247
	GS5	619	13	8	0.324	0.0030	0.0033	-0.231	-0.662	-0.600
	Pid3	667	21	16	0.654	0.0047	0.0050	-0.165	-4.603**	-3.382**
	SAP8	670	3	4	0.287	0.0004	0.0007	-0.573	-2.378*	-2.121
	Ehd1	482	5	5	0.317	0.0026	0.0016	1.031	0.946	1.165
	Xa23	649	7	9	0.722	0.0022	0.0017	0.593	0.109	0.337
	Average	616.7	9	8	0.437	0.0023	0.0023	0.040	0.036	-0.293
GZ	*SKC1*	613	6	9	0.684	0.0021	0.0014	0.932	0.985	1.166
	GS5	619	13	15	0.558	0.0058	0.0030	2.063	-0.092	0.903
	Pid3	667	21	21	0.576	0.0041	0.0045	-0.239	-3.415**	-2.553*
	SAP8	670	4	5	0.491	0.0008	0.0009	-0.153	-0.593	-0.532
	Ehd1	482	6	7	0.534	0.0042	0.0018	2.443*	-0.152	0.927
	Xa23	649	9	12	0.733	0.0025	0.0020	0.486	1.191	1.127
	Average	616.7	9.8	11.5	0.596	0.0033	0.0023	0.618	0.268	0.718
NP	*SKC1*	613	8	8	0.650	0.0022	0.0019	0.267	1.132	0.987
	GS5	619	13	11	0.582	0.0031	0.0031	0.053	-1.572	-1.144
	Pid3	667	13	21	0.655	0.0046	0.0028	1.126	-0.688	0.026
	SAP8	670	5	6	0.511	0.0008	0.0011	-0.479	-2.829*	-2.421*
	Ehd1	482	11	8	0.575	0.0044	0.0033	0.719	-3.599**	-2.378*
	Xa23	649	9	15	0.744	0.0027	0.0020	0.699	1.196	1.225
	Average	616.7	9.8	11.5	0.620	0.0030	0.0024	0.398	0.017	0.273
WR	*SKC1*	613	4	5	0.470	0.0011	0.0015	-0.609	-0.051	-0.256
	GS5	619	12	12	0.643	0.0043	0.0045	-0.154	0.443	0.292
	Pid3	667	10	10	0.610	0.0031	0.0035	-0.298	0.813	0.536
	SAP8	670	2	3	0.354	0.0005	0.0007	-0.406	-0.843	-0.829
	Ehd1	482	8	7	0.720	0.0029	0.0039	-0.716	1.309	0.783
	Xa23	649	5	6	0.738	0.0037	0.0018	2.736	1.112	1.880*
	Average	616.7	6.8	7.2	0.589	0.0026	0.0026	0.092	0.464	0.105

S：Number of polymorphic (segregating) sites；h：Number of haplotypes；Hd：Haplotype (gene) diversity；π：Nucleotide diversity；Theta-w：Watterson's parameter for silent sites；D: Tajima's D；D* and F*： Fu and Li's D* and Fu and Li's F* respectively. P**<0.02.

Supplementary Table S6 Haplotype frequency of six sequenced genes of KSR, GZ, NP and WR groups

Population	*Ehd1*	*GS5*	*Pid3*	*SAP8*	*SKC1*	*Xa23*	Average
KSR	21.4%	21.0%	21.2%	21.3%	21.3%	21.4%	21.3%
GZ	39.1%	38.3%	39.4%	39.1%	39.0%	39.1%	39.0%
NP	36.9%	36.4%	36.8%	36.8%	37.0%	36.7%	36.8%
WR	2.5%	2.0%	2.6%	2.8%	2.7%	2.8%	2.6%
