# Supplementary material for: Kam Sweet Rice (Oryza sativa L.) Is a Special Ecotypic Rice in Southeast Guizhou, China as Revealed by Genetic Diversity Analysis
Source: Front Plant Sci. 2022 Mar 7;13:830556. doi: 10.3389/fpls.2022.830556 (PMC8940365; doi:10.3389/fpls.2022.830556)
Supplement: Supplementary file 2 [file Data_Sheet_2.docx]

**Overview of Kam Sweet Rice**

Guizhou province is rich in rice landrace resources, ranking fourth in the number of landraces in China, after Guangxi, Guangdong, and Yunnan provinces (Han and Cao 2005; Ruan et al., 2007). The traditional rice variety "He" Resources are cultivated by the local Dong people for thousand years in southeast Guizhou, among them, “glutinous-He” varieties accounted for more than 90% (Chen and Deng, 2013; Luo, 2014).Scholars defined it as a traditional rice group, which originated from the complex and diverse ecological environment in southeast Guizhou and the traditional farming system of the Dong people, and was formed through long-term natural evolution and artificial selection. It is not a biological taxonomic unit, but is an original, ecological rice landrace (Ma, 1979). “Xiang he nuo” is the common name of Dong people for “HE” resources. (“xiang” means fragrant, “he” means HE resource, “nuo” means a kind of glutinous rice).

Richard Stone, chief editor of *science*, translates “Xiang he nuo” as “Kam Sweet Rice (KSR)” (Dong people are also called Kam People) (Stone, 2008), and the Food and Agriculture Organization (FAO) called it a “Specialty Rice” (Bedigian, 2003). The Dong people named KSR varieties by using seven elements, including diverse traits, growth environment, different stages of maturity, origin, the name of person who bred or cultivated KSR, quality and harvest way (Pan and Long, 2013).

**Ethnobiology study and KSR collection**

We used the method of "literature review - field investigation - farmer interview - sample collection” to collect KSR from 2013 to 2015. The biological and other remarkable characteristics of these KSR have been identified and recorded in collection (Wang et al, 2018).

Ethnobiology studies were conducted in Dong villages in southeast Guizhou. Through the snowball technique, a large number of respondents (Dong farmers) were interviewed in Dong villages, including key informant and semi-structured interviews. Survey sites include but are not limited to farmers' homes, farmland, fish ponds, streets, and workshops. Key informant interviews were conducted with mainly local experts, village cadres, clan elders (village elders, headmen, etc.) and inheritors of intangible cultural heritage. The semi-structured interviews involved open-ended questions and conversations with informants in the above scenes. All interview procedures involved were in accordance with the International Society of Ethnobiology Code of Ethics, including procuring prior informed consent before interviews.

**References**

Bedigian D. Specialty rices of the world. Breeding, production and marketing. Econ Bot. 2003; 57(1):160. [https://doi.org/10.1663/0013-0001(2003)057[0160:BR]2.0.CO;2](https://doi.org/10.1663/0013-0001(2003)057%5b0160:BR%5d2.0.CO;2).

Chen X.L., Deng MW. (2013)Research on ecological culture of Dong nationality in China. Beijing: China Forestry Publishing House.

Han, L.Z., and Cao, G.L. (2005). Status of Collection, Conservation and Propagation of Rice Germplasm in China. Journal of Plant Genetic Resources. 6(3): 359-364.

Luo K.Z. (2014) Preservation and Innovation: Adapting tradition to modern in Huanggang Dong Village of Liping County. Beijing: Nationalities publishing house.

Ma H.L. (1979) The production problem of He and He area, He and agriculture production. Guiyang: Guizhou crop germplasm resource office; 1-10.

Pan Y.R., Long X. Y. (2013) Nomenclature and Classification of Kam Sweet Rice Varieties: A Case

Study of Agricultural Cognition of China’s Mountain Peoples. Journal fo Original Ecological National Culture. 5(1): 91-98.

Ruan, R.C., Chen, H.C., You, J.M., Zhu, Y.Q., Zhu, M., and Chen, N.G. (2007). Current Status and Prospects of Conservation for Rice Genetic Resources in Guizhou. Seeds. 26(10):53-56. doi: 10.3969/j.issn.1001-4705.2007.10.017

Stone R. Intellectual property: Chinese province crafts pioneering law to thwart biopiracy. Science. 2008; 320(5877):732–733. <https://doi.org/10.1126/science.320.5877.732>.

Wang, Y.J., Jiao, A.X., Chen, H.C., Ma, X.D., Cui, D., Han, B., et al., (2018). Status and factors influencing on-farm conservation of Kam Sweet Rice (Oryza sativa L.) genetic resources in southeast Guizhou Province, China. Journal of Ethnobiology and Ethnomedicine. 14:76.doi: 10.1186/s13002-018-0256-1
